# Implementation and sustainment of diverse practices in a large integrated health system: a mixed methods study

**DOI:** 10.1186/s43058-020-00053-1

**Published:** 2020-07-03

**Authors:** Andrea L. Nevedal, Caitlin M. Reardon, George L. Jackson, Sarah L. Cutrona, Brandolyn White, Allen L. Gifford, Elizabeth Orvek, Kathryn DeLaughter, Lindsay White, Heather A. King, Blake Henderson, Ryan Vega, Laura Damschroder

**Affiliations:** 1 Center for Innovation to Implementation, VHA Palo Alto Health Care System, 795 Willow Road (152-MPD), Menlo Park, CA 94025 USA; 2 Center for Clinical Management Research, VHA Ann Arbor Healthcare System, 2215 Fuller Rd., 152, Ann Arbor, MI 48105 USA; 3 Durham Center of Innovation to Accelerate Discovery and Practice Transformation (ADAPT), Durham VHA Health Care System, HSR&D (152) Suite 600, 411 West Chapel Hill Street, Durham, NC 27701 USA; 4 Department of Population Health Sciences and Division of General Internal Medicine, Duke University School of Medicine, 215 Morris Street, Durham, NC 27701 USA; 5 Center for Healthcare Organization & Implementation Research, Bedford & Boston VHA Medical Centers, 200 Springs Road (152), Building 70, Bedford, MA 01730 USA; 6 Department of Population and Quantitative Health Sciences, University of Massachusetts Medical School, 368 Plantation Street, The Albert Sherman Center, Worcester, MA 01605 USA; 7 Section of General Internal Medicine & Department of Health Law, Policy & Management, Boston University, 715 Albany St., Talbot Building, T2W, Boston, MA 02118 USA; 8 Diffusion of Excellence, VHA Innovation Ecosystem, 810 Vermont Avenue NW, Washington, DC, 20420 USA; 9 VHA Office of Discovery, Education and Affiliate Networks, 810 Vermont Avenue NW, Washington, DC, 20420 USA

**Keywords:** Consolidated Framework for Implementation Research (CFIR), Qualitative methods, Model of diffusion, Sustainability, Learning health system, Veterans, Veterans Health Administration (VHA), VHA Innovation Ecosystem, VHA Diffusion of Excellence

## Abstract

**Background:**

One goal of health systems seeking to evolve into learning health systems is to accelerate the implementation and sustainment of evidence-based practices (EBPs). As part of this evolution, the Veterans Health Administration (VHA) developed the Innovation Ecosystem, which includes the Diffusion of Excellence (DoE), a program that identifies and diffuses Gold Status Practices (GSPs) across facilities. The DoE hosts an annual “Shark Tank” competition in which leaders bid on the opportunity to implement a GSP with 6 months of implementation support. Over 750 diverse practices were submitted in cohorts 2 and 3 of Shark Tank; 23 were designated GSPs and were implemented in 31 VA networks or facilities. As part of a national evaluation of the DoE, we identified factors contributing to GSP implementation and sustainment.

**Methods:**

Our sequential mixed methods evaluation of cohorts 2 and 3 of Shark Tank included semi-structured interviews with at least one representative from 30/31 implementing teams (*N* = 78/105 people invited) and survey responses from 29/31 teams (*N* = 39/47 invited). Interviews focused on factors influencing implementation and future sustainment. Surveys focused on sustainment 1.5–2 years after implementation. The Consolidated Framework for Implementation Research (CFIR) informed data collection and directed content analysis. Ordinal scales were developed inductively to rank implementation and sustainment outcomes.

**Results:**

Over 50% of teams (17/30) successfully implemented their GSP within the 6-month implementation period. Despite extensive implementation support, significant barriers related to centralized decision-making, staffing, and resources led to partial (*n* = 6) or no (*n* = 7) implementation for the remaining teams. While 12/17 initially successful implementation teams reported sustained use of their GSP, over half of the initially unsuccessful teams (*n* = 7/13) also reported sustained GSP use 1.5 years after the initial implementation period. When asked at 6 months, 18/27 teams with complete data accurately anticipated their future sustainability based on reported sustainment an average of 1.5 years later.

**Conclusions:**

Most teams implemented within 6 months and/or sustained their GSP 1.5 years later. High levels of implementation and sustainment across diverse practices and teams suggest that VHA’s DoE is a successful large-scale model of diffusion. Team predictions about sustainability after the first 6 months of implementation provide a promising early assessment and point of intervention to increase sustainability.

Contributions to the literature

- Examples of system-level structures and processes to identify, diffuse, and sustain best practices are rare; the VHA DoE can serve as a model of diffusion for other large learning health systems.
- Our findings indicate that implementation timelines may be arbitrarily set and that failure to meet pre-specified implementation milestones does not necessarily hinder sustainment.
- Research on sustainability is nascent; our results indicate that teams’ anticipated sustainment after initial implementation may be a useful assessment and present a fruitful point of intervention when teams do not expect to sustain their practice.

## Background

Implementation science is the systematic study of methods to encourage the integration of evidence-based practices (EBPs) into routine care to improve outcomes [1, 2]. EBPs include practices that are supported by sufficient evidence from research studies, clinical experience, and/or patient values and preferences [1, 3]. Implementation is the means by which an EBP is assimilated into an organization and usually a deliberately initiated process, where individuals aim to bring EBPs into routine use as designed [4, 5]. Despite established effectiveness and despite implementation efforts, most EBPs are not rapidly implemented or sustained in health systems, delaying or halting benefits to patients, employees, and systems [1, 6–12]. As a result, health systems are seeking to evolve into learning health systems, with one goal to support continuous learning and innovation. Though learning systems have successfully improved health care quality and efficiency [13], knowledge is only just emerging about how learning health systems may accelerate identification, diffusion, and sustainment of multiple EBPs across systems.

The Veterans Health Administration (VHA), the largest integrated health system in the USA, seeks to evolve into a learning health system [8, 10, 14–16]. As part of this evolution [17, 18], the VHA developed the Innovation Ecosystem, which aims to embed innovation in the core fabric of the VHA, build a collaborative innovation community, and deliver a repeatable process for scaling innovation [19]. The VHA Innovation Ecosystem includes two programs: the Innovators Network (iNET) and the Diffusion of Excellence (DoE). The iNET trains employees on innovation-related competencies and provides support for an innovation development pathway [20]. The focus of this evaluation is on the DoE.

The DoE program is guided by a 5-phase lifecycle designed to shepherd Gold Status Practices (GSPs) from early piloting to national diffusion [8, 10, 14–16, 21]. In *phase 1*, VHA employees develop and implement innovative practices in their local facility. These innovations may or may not be supported by a wide array of VHA funding sources and support. If the innovation is successfully implemented with measurable positive impact for the system, employees, or patients, then the lead developer (a VHA employee or team) can submit their practice for consideration in phase 2. In order to be considered, innovations must align with one of five VHA high priority areas: access, care coordination, employee engagement, quality and safety, or veteran experience.

The central function of *phase 2* includes a “Shark Tank” process that selects GSPs from applications that have completed phase 1. A governance board, comprising national executive-level VHA leaders, reviews applications and approves a shorter list of finalists who are entered into a Shark Tank competition. Finalists develop a 5-min pitch video describing their innovation and the resources required to implement and use routinely. VHA facility or network directors, who volunteer to participate as a “Shark” in the Shark Tank, review pitch videos and place bids for one or more practices that they want to implement in their facility/network. Winning bidders receive 6 months of external implementation support. Bids typically include key resources (e.g., staff, office space, travel funds) that will be provided if their bid is selected. The DoE governance board reviews bids and selects a final list of practices that are designated GSPs. GSPs have a wide range of evidence supporting them. Some GSPs have emerging evidence based on system, employee, or patient experience within one facility (e.g., a workflow management system that addresses issues with the process for authorizing artificial limbs for patients) or may have published research evidence from clinical trials conducted by VHA and/or non-VHA researchers (e.g., an oral care practice to prevent non-ventilator-associated pneumonia among inpatients) [22].

In *phase 3*, GSPs are implemented into facilities with the winning Shark Tank bids. As of June 2020, 5 Shark Tanks have been conducted, with 5 cohorts of GSPs. Each facility identifies an “Implementing Fellow,” a VHA employee who leads GSP implementation at their facility/network. The Implementing Fellow attends a 2-day in-person “Diffusion Base Camp” to meet with Gold Status Fellows (the VHA employee who developed the GSP) and an Implementation Support Provider (a VHA staff member or contractor) who will provide external project management expertise, coordinate weekly meetings, and help track tasks and milestones. During Base Camp, GSP teams (Gold Status Fellow, Implementing Fellow, and Implementation Support Provider) attend plenary sessions focused on helping to develop implementation strategies. Teams also work together to develop a plan to implement their GSP over the next 6 months. After the 6-month implementation phase, teams reconvene for a final meeting to discuss implementation experiences and lessons learned to encourage wide dissemination and to inform implementation at additional sites.

In *phase 4*, the DoE governance board selects a subset of GSPs to receive national-level support for broader diffusion. All GSPs, including those not chosen for more formal support, may nonetheless diffuse organically (i.e., other facilities may decide to implement a GSP on their own). To help promote organic diffusion, the DoE created an online marketplace that allows VHA leaders and employees to learn about GSPs. In *phase 5*, the DoE governance board selects GSPs that were highly successful during initial diffusion to receive support for national diffusion.

The aim of this study was to identify factors contributing to successful implementation and sustainment of 23 diverse GSPs from the 2nd and 3rd Shark Tank cohorts. We have partnered with national DoE leaders to conduct this evaluation. These results have been used to guide refinement of the DoE as a model of diffusion and support the evolution of VHA as a learning health system.

## Methods

**Fig. 1:**
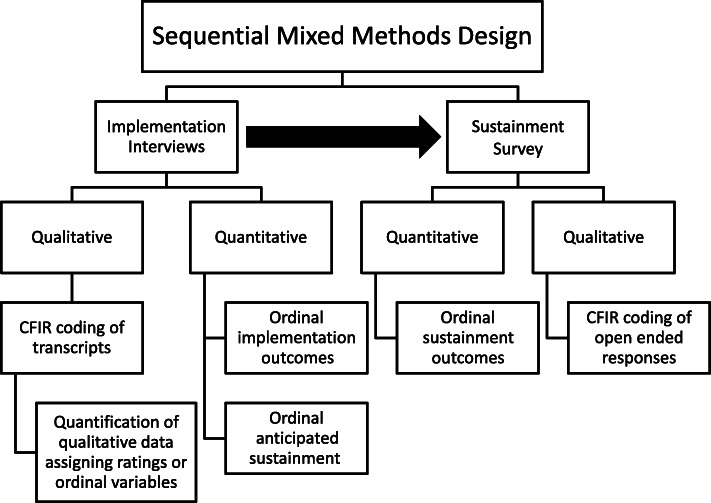
Sequential mixed methods design

This was a sequential mixed methods evaluation (Additional file 1. Consolidated criteria for reporting qualitative studies (COREQ): 32-item checklist) [23]. Figure 1 lists the qualitative and quantitative sequence and data used in this evaluation. The research team was embedded within VHA and partnered closely with the DoE to conduct the national evaluation of DoE. The evaluation team provided high-level, rapid feedback and recommendations between each Shark Tank cohort to help DoE leaders strengthen program infrastructure (e.g., Shark Tank application process). Per regulations outlined in VHA Program Guide 1200.21, this evaluation has been designated a non-research quality improvement activity that was ethics exempt.

### Sample

The team used purposeful criterion sampling and snowball sampling [24] to recruit implementation team members from cohorts 2 and 3 of Shark Tank. Implementation Support Providers (implementation support/project management) and Implementing Fellows (implementation leader at the VHA facility/network) were interviewed first because they were most closely involved with GSP implementation and were our main points of contact. Using snowball sampling, Implementing Fellows identified additional staff members at their facility/network who were involved in implementation to participate in interviews. Participants were invited via email to participate in a semi-structured telephone interview. The same individuals were also emailed a sustainment survey, except for Implementation Support Providers, who were only involved during the 6-month implementation period.

### Data collection

#### Implementation interviews

Telephone interviews were conducted from June to September 2017 and February to April 2018, after the 6-month implementation period ended for cohorts 2 and 3, respectively. A PhD-level medical anthropologist and qualitative methodologist (AN) and an MPH-level qualitative analyst (CR) conducted interviews that lasted approximately 60 min. To boost rapport and accuracy of responses, interviewers encouraged participants to provide candid responses about their experiences with DoE. Participants were also informed that the evaluation team was not affiliated with DoE. Interviews were audio-recorded and transcribed with participant consent. The purpose of the interviews was to understand contextual factors associated with implementation outcomes, assess overall implementation success, and discuss prospects for sustaining their GSP. The interview guide (Additional file 2) was informed by the Consolidated Framework for Implementation Research (CFIR) [5, 25, 26], which defines constructs across five domains of potential influences on implementation. The interview guide was pilot tested and vetted by an interdisciplinary team with implementation science and qualitative methods expertise.

#### Sustainment surveys

Sustainment surveys were emailed in June 2019, 14–21 months after the initial 6-month implementation period ended, an average of 1.5 years. The purpose of the survey was to assess the level of GSP sustainment as well as understand contextual factors associated with GSP sustainment (Table 1).

**Table 1:** Sustainment survey questions

1. Was the **[Gold Status Practice]** successfully implemented at your facility?^a^ a. Yes/No 2. Is the **[Gold Status Practice]** still being **[used/done]** at your facility? a. Yes/No a. Why or why not? 3. Have there been any changes or adaptations to the **[Gold Status Practice]**? a. Yes/No b. Why or why not? 4. Please describe any measures you may use to track **[Gold Status Practice]** effectiveness a. Measure(s): b. Description of Measure(s): What is the data source? How is the measure computed?	

^a^Question only asked to teams with partial or no implementation

## Data analyses

### Implementation interviews

#### Coding data

Transcripts were de-identified and then uploaded into Dedoose [27], a collaborative qualitative software program. The researchers conducted directed content analysis [28] by iteratively developing a codebook (Additional file 3) using deductive codes derived from CFIR constructs and inductive codes for additional categories grounded in the data, including sustainability prospects and relationships between codes [5]. Using a rigorous consensus-based coding process [29], two researchers (CR, AN) independently coded transcripts and met weekly to discuss and resolve discrepancies.

#### Aggregating data

The researchers exported coded data from Dedoose into a Microsoft Word memo template; the template facilitates aggregating, summarizing, and rating data. One memo was created for each GSP implementation team. The memo was organized by CFIR constructs.

#### Rating data

The researchers (AN and CR) alternated writing memos summarizing and rating each CFIR construct per facility/network [25, 26]. Ratings were used to ascertain the salience of barriers and facilitators and facilitate comparisons across constructs and implementation teams. Ratings were based on the strength of manifestation (strong or weak influence on implementation) and valence (positive or negative influence on implementation). Ratings ranged from + 2 to − 2, including 0 for neutral; missing data (M) and mixed influences (X) were also included. The alternate researcher (AN or CR) reviewed the memo and used track changes to indicate disagreements. The researchers met weekly to discuss and resolve discrepancies and note emerging patterns in the data.

### Sustainment surveys

Survey responses were imported into MS Excel and aggregated using descriptive statistics. When applicable, the researchers coded open-ended responses using the CFIR codebook.

### Data interpretation

#### Implementation outcomes

We used qualitative interview data to assign an ordinal value to indicate implementation success at the end of the 6-month implementation period; the scale was adapted from the criteria developed by the Institute of Healthcare Improvement’s Breakthrough Series Collaborative Model Progress Scale (see Table 2) [30, 31].

**Table 2:** Definitions for implementation and sustainment outcomes for Gold Status Practices (GSP)

**Implementation outcomes at 6 months**	**Definition**
Implemented	Teams who implemented all GSP components and achieved critical milestones.
Partially implemented	Teams who implemented some GSP components and achieved some but not all milestones.
Not implemented	Teams who completed initial implementation planning but did not implement any GSP components and did not achieve any critical milestones.
**Anticipated sustainment outcome at 6 months**	**Definition**
Sustainment anticipated	Teams who anticipated sustaining all GSP components
Sustainment challenges anticipated	Teams who anticipated challenges might hinder sustainment of GSP components
**Sustainment outcomes at approximately 1.5 years**	**Definition**
Sustained	Teams who sustained all GSP components
Partially sustained	Teams who sustained some GSP components
Not sustained	Teams who did not sustain any GSP components

#### Aligning CFIR constructs and implementation outcomes

After completing memos, the researchers copied qualitative summaries and quantitative ratings into a MS Excel matrix template: the columns were organized by implementation team (and ordered by implementation outcome) and the rows were organized by CFIR construct (and inductively identified codes). This allowed the team to conduct matrix [32, 33] analyses to align and compare qualitative and quantitative data and to perform case and construct analysis. We performed case analyses to describe how contextual barriers and facilitators influenced implementation outcomes for each GSP implementation team. Construct analyses allowed the team to determine which CFIR constructs were linked to implementation outcomes.

#### Anticipated sustainment and sustainment outcomes

We assessed anticipated sustainment immediately after the initial 6-month implementation period ended using an ordinal scale; ratings were developed inductively based on responses to interview questions (see Table 2). Using a similar process for survey responses, we assessed sustainment outcomes 14–21 months after the initial 6-month implementation period (an average of 1.5 years).

#### Aligning sustainment outcomes with implementation outcomes and CFIR constructs

The team incorporated sustainment outcomes into the matrix to explore linkages between sustainment outcomes, implementation outcomes, team members’ expectations for sustainment when asked at 6 months, and qualitative data with quantitative ratings by CFIR construct.

## Results

Over 750 diverse practices were submitted across cohorts 2 and 3; 23 were designated as a GSP [10, 14]. Additional file 4 describes each GSP. Implementation interviews were completed with 78 (*n* = 105 invited) implementation team members, including 7 Implementation Support Providers, 31 Implementing Fellows, and 40 other staff involved with implementation. Implementing Fellows from 22/23 GSPs and 30/31 teams from cohorts 2 and 3 participated in an interview; there were 31 teams because some GSPs were implemented by more than one team at more than one facility/network—thus, there were more teams than GSPs. Implementation Support Providers worked with Implementing Fellows to provide implementation support and project management. Implementing Fellows led implementation at their local facility and held VHA positions ranging from entry-level to service-line chief. Other staff were VHA employees who assisted Implementing Fellows throughout the implementation and held positions ranging from entry-level to facility director. The following sections describe initial implementation, anticipated sustainment, and sustainment outcomes for multiple EBPs to provide evidence to understand DoE as a model of diffusion.

### Initial implementation

Over 50% of teams (*n* = 17 out of 30) successfully implemented their GSP within the 6-month implementation period; the remaining teams had partial (*n* = 6) or no (*n* = 7) implementation. In the following sections, we describe our results with the associated CFIR construct(s) which are italicized (see Table 3 for CFIR construct definitions). *External Policies and Incentives* received negative ratings across most teams, indicating a general barrier to implementation. *External Change Agents* received positive ratings across teams regardless of the implementation outcome, reflecting broad appreciation for this support. *Engaging Key Stakeholders* and *Available Resources* received ratings that varied based on implementation outcomes. Ratings for these CFIR constructs are provided in Additional file 5.

**Table 3:** Definitions for the Consolidated Framework for Implementation Research (CFIR) constructs

External Change Agents	Individuals from outside the organization who formally facilitate implementation
External Policies and Incentives	External policy, regulations, and mandates
Engaging Key Stakeholders	Individuals from within the organization that are directly impacted by the innovation
Available Resources	Resources for implementation and on-going operations of the innovation
Complexity	Complexity of the innovation
Compatibility	Fit between the innovation and existing workflows and systems
Relative Priority	Importance of implementation within the organization
Organizational Incentives and Rewards	Extrinsic incentives such as goal-sharing awards, performance reviews, promotions, and raises in salary

#### *External Policies and Incentives*: construct rated negatively across teams

*External Policies and Incentives* received negative ratings, regardless of the implementation outcome, because this was the source of delays for many teams during implementation. *External Policies and Incentives* is a broad construct that includes centralized decision-making processes (i.e., external policies, approvals, and procedures affecting implementation). For example, policies and procedures that led to delays in hiring necessary new staff, purchasing supplies, or obtaining approvals to software or websites. Teams with successful implementation also experienced barriers related to centralized decision-making, but were able to resolve these types of challenges to achieve their milestones within the 6-month implementation period:

We kept knocking on the door saying we need this. Not only for just this project but for operating the website for our facility. We would just keep knocking on the door and just didn’t take no for an answer. [Implemented, Team #9/Cohort 2]

Teams who did not implement or only partially implemented (hereafter: unsuccessful implementation) worked hard to resolve challenges related to centralized decision-making, but they were insurmountable within the 6-month implementation period. For example, one participant described how the national hiring freeze in 2016 delayed posting new job positions needed to support the GSP (*External Policies and Incentives* hindered *Engaging Key Stakeholders*):

Although this position itself was not subject to [the hiring freeze], all the support staff that we had in house with hiring [were affected], so it got kind of pushed to the side a bit. […] And so, once we got the functional statement done, then we had to advertise the job and interview. Well there was a complication with the way the job was posted or advertised through the Human Resources system, and we got very few candidates […] So we decided we should go back out and look again. And so, Human Resources reposted the job in a different way and then we had to set up the interview panel. [Not Implemented, Team #2/Cohort2]

#### *External Change Agents*: construct rated positively across teams

Implementation Support Providers and Gold Status Fellows (*External Change Agents*) received positive ratings, regardless of the implementation outcome, due to the guidance and encouragement they provided throughout the initial 6-month implementation period. Gold Status Fellows developed the GSP and shared their content expertise by describing implementation experiences from their facilities, training teams, and providing informational materials:

[The Gold Status Fellow] had [all the information and materials] for us and gave it to us on a silver platter. [Not Implemented, Team #7/Cohort 2]

Implementation Support Providers contributed project management expertise, including coordinating weekly meetings and tracking tasks and milestones. In addition, they acted as liaisons between implementation teams and local- and national-level VHA leaders as needed:

[The Implementation Support Providers] were great in terms of facilitating, keeping things on track, keeping checklists, making sure everybody was doing what they needed to do and knew what was expected. Always sort of the ‘eyes on the prize.’ And the biggest thing is advocating for us to our leadership about what we need. It’s so nice to have a voice, I talk every day until I’m blue in the face about what we need, and sometimes you just get that learned helplessness, and you stop doing it. But it’s so nice when it comes from somebody else. We were able to secure an extra printer copier fax; that may not sound like a big deal, but to us it was huge. [Implemented, Team #3/Cohort3]

Gold Status Fellows and Implementation Support Providers [*External Change Agents*] also conducted site visits to help engage and train facility staff:

The point where engagement really caught on was when the [External Change Agents] came to our site for a 2-day sort of in-service on the process […] When [our staff] saw these people in flesh and blood, and saw what they were doing, and saw how passionate they were about it, that’s when buy-in happened. [Implemented, Team #3/Cohort3]

Gold Status Fellows and Implementation Support Providers provided valuable support which helped most teams complete rapid implementation; teams were only unsuccessful in the face of insurmountable barriers at the facility/network level, which are described below.

#### *Engaging Key Stakeholders*: construct ratings associated with implementation outcomes

Ratings for *Engaging Key Stakeholders* were more positive for successful implementations compared to less successful implementations. Successful teams had the necessary staff in place (or staff that could temporarily fulfill roles) prior to implementation. Implementing Fellows from successful teams also excelled in obtaining buy-in from key staff:

We did have a great group of [staff]. We may not have had many, but we had a great group of [staff] who were willing to help me. [Implemented, Team #9/Cohort 2]

Conversely, unsuccessful implementation teams did not have necessary staff in place (*Engaging Key Stakeholders*):

Those [staff] positions have been approved, but I do not know if they have been posted at this point. You have to have the staff before you can implement. [Not Implemented, Team #1a/Cohort 3]

#### *Available Resources*: construct ratings associated with implementation outcomes

Successful teams more often had the necessary resources *(Available resources)* in place prior to implementation:

We have all the tools. We have [software]. We have VistA [the VHA information system that includes the electronic health record]. [Implemented, Team #2a/Cohort 3]

In contrast, teams who did not achieve implementation milestones often did not have dedicated physical space, equipment, software, supplies, or funding during the implementation period. For example, an Implementing Fellow was determined to find alternate funding sources to support an underfunded GSP, but it was not the right type of funding, and it could not be used to support the GSP (*External Policies and Incentives* hindered obtaining *Available Resources*):

We ran into an issue with…locating funding for the program. […] We just beat the ground, and still are beating the ground to try to just get funds. [DoE] offered to provide funds, but we ran into a fiscal issue. [Not Implemented, Team #10/Cohort 3]

#### Sustainment

The following sections describe results from anticipated sustainment outcomes, sustainment outcomes at 1.5 years, and implementation outcomes versus both sustainment outcomes.

#### Anticipated sustainment

At the end of the 6-month initial implementation period, 30 teams were asked during their interview, how likely they would continue to use their GSP; all but one team provided sufficient responses to this question. Most teams (*n* = 22/29 with complete data; 76%) anticipated that their GSP would be sustained; these teams included 11 of the 13 who did not achieve initial implementation milestones (Table 4 compares implementation outcomes and anticipated sustainment). These latter teams believed that though delayed, they would achieve their milestones after the initial 6-month period and then go on to sustain their practice; they simply needed a longer implementation timeline to overcome barriers. However, some teams (*n* = 7/29; 24%) did foresee challenges that would potentially continue to hinder implementation and/or sustainment of their GSP. These teams were especially concerned about maintaining protected time for staff, training new staff, and/or retaining trained staff (*Engaging Key Stakeholders*), and they described challenges related to the high level of dedicated time needed to deliver the GSP (*Complexity; Compatibility*), other competing priorities (*Relative Priority*), and an inability to track use or impact of the GSP because of lack of adequate measures (*Organizational Incentives and Rewards*).

**Table 4:** Implementation outcomes versus anticipated sustainment^a^

Team implementation outcomes at 6 months	Team anticipated sustainment		
	Sustainment anticipated	Sustainment challenges anticipated	Total
Implemented	11	5	16
Partially implemented	5	1	6
Not implemented	6	1	7
Total	22	7	29^a^

^a^Two teams had missing data

#### Reported sustainment 1.5 years later

**Fig. 2:**
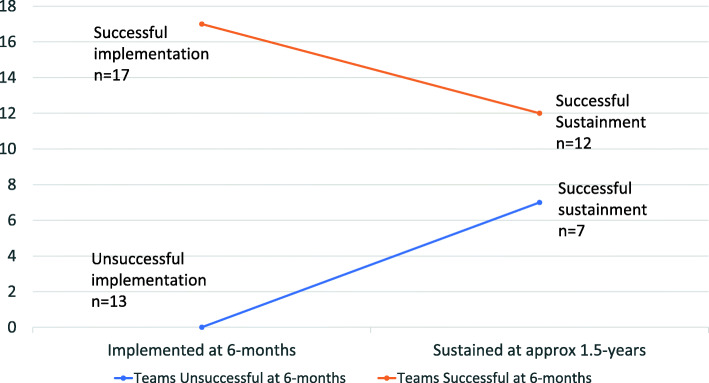
Comparing implementation and sustainment outcomes

Sustainment surveys were completed by 39 (*n* = 47 invited) implementation team members, including 18 Implementing Fellows and 21 other staff. Implementation Support Providers were not surveyed because they were not involved during the sustainment phase. At least one representative from all the 23 GSPs and from 28 of 31 teams completed the survey. Over two-thirds of teams (*n* = 19/28 with complete data) reported sustained use of their GSP 1.5 years after the implementation period ended. When comparing implementation and sustainment outcomes (Table 5 and Fig. 2), 12 out of 17 teams who implemented in 6 months sustained their GSP 1.5 years later. In contrast, 7 out of 11 teams who were unsuccessful at implementation in 6 months completed implementation and sustained their GSP 1.5 years later. Among the nine teams who did not report sustainment, many responded to the open-ended survey question by describing barriers related to losing necessary staff (*Engaging Key Stakeholders*) or lack of necessary resources (*Available Resources*) during the 1.5 years following initial implementation. Challenges related to staffing included turnover among the Implementing Fellows or clinicians responsible for the GSP, inadequate GSP training for new staff members, or other staff remaining unaware of the GSP. For example, one response stated the GSP was partially in use, but they were struggling to engage additional staff:

Our trainees who were using [the GSP] can reasonably be said to be sustaining use. Other providers have access to [the GSP] without additional training – so we cannot be sure of how/why they’re using it. As a training hospital, residents who have cycled out of rotation will have fallen out of our utilization audience." [Partially Implemented, Team #4/Cohort 3].

**Table 5:** Implementation outcomes versus sustainment outcomes^a^

Team implementation outcomes at 6 months	Team sustainment outcomes 1.5 years later			
	Sustained	Partially sustained	Not sustained	Total
Implemented	12^a^	3	2	17
Partially implemented	3	1^a^	1	5
Not implemented	4	2	0^a^	6
Total	19	6	3	28

^a^Two-way concordance of ratings/responses

Inadequate *Available Resources* or low *Relative Priority* were other challenges leading to non-sustainment. Some teams intended to continue their GSP but were currently on a hold for unexplained reasons. One team reported replacing the original GSP with a different, though similar practice, because it required fewer resources to deliver. Overall, barriers that explained non-sustainment were similar to those encountered during implementation.

#### Comparing implementation outcomes, anticipated sustainment, and sustainment outcomes

Twenty-seven out of thirty teams had complete data for implementation outcomes, anticipated sustainment, and sustainment outcomes (Table 6). Over half of teams (9/16, 56%) that were successful within the initial 6-month implementation period were accurate in anticipating future sustainment. For example, one team member said:

Absolutely [sustain the GSP]. […] My boss has to report to leadership, so she is running data quarterly to make sure people are using it. [Implemented, Team #8/Cohort 3]

**Table 6:** Comparing implementation outcomes, anticipated sustainment, and sustainment outcomes of Gold Status Practices^a^)

Implementation outcomes at 6 months	Anticipated sustainment^b^		Sustainment outcomes at 1.5 years^c^			Did anticipated sustainment align with reported sustainment?^d^		
	Sustainment anticipated	Significant challenges anticipated	Sustained	Partially sustained	Not sustained	Yes	No	Total
Implemented (*n* = 16)	11^e^	5	11^e^	3	2	9	7	16
Partially implemented (*n* = 5)	4	1^e^	3	1^e^	1^e^	4	1	5
Not implemented (*n* = 6)	5	1^e^	4	2^e^	0^e^	5	1	6
Total	20	7	18	6	3	18	9	27

^a^4 teams had missing data

^b^Based on open-ended responses to the question, “How likely is it that the practice will continue to be used at your site?” at the end of the 6-month initial implementation period

^c^Based on responses to emailed survey asking, “Is the Gold Status Practice still used at your facility? Why/why not?” 1.5 years after initial implementation period

^d^Based on comparison of anticipated sustainment and reported 1.5-year sustainment. Coded as yes, if anticipated sustainment AND reported sustained use. Coded as no, if significant challenges anticipated AND reported 1.5-year partial sustainment or not sustained

^e^Two-way concordance of ratings/responses

1.5 years later, this individual reported sustained use of the GSP:

[The GSP] has been a useful tool at our site. It helps manage the workflow. This is checked on a weekly basis. [Sustained, Team #8/Cohort 3]

Conversely, five initially successful teams anticipated challenges to sustaining their GSP because of staff turnover or decreasing use by employees; two of these teams accurately anticipated significant challenges and reported partial or no sustainment 1.5 years later. Anticipating challenges, one team said:

We’re in the process of planning our next one [GSP event]. [However], I don’t know if we have enough [external partners with resources] to support it. [Implemented, Team #7/Cohort 3]

1.5 years later, the participant reported that the GSP:

Hasn’t been done since June 2018, but we plan to continue [working on it]. [Not Sustained, Team #7/Cohort 3]

Compared to initially successful teams, those who were not successful were more often accurate (9/11, 81%) about anticipated sustainment; 7 accurately predicted sustainment and 2 accurately predicted challenges. For example, one team anticipated achieving their implementation milestones late and then sustained use:

The Committee just approved my [employees] to be the [involved with the GSP]. […] I think it’ll help with sustainment, to make sure that we continue using the [GSP]. [Partially Implemented, Team #6/Cohort 2].

In response to the sustainment survey 1.5 years later, the same individual confirmed sustainment:

Yes [the GSP is sustained], the program helps with employees’ development, consistency with skills, and adds uniformity. [Sustained, Team #6/Cohort 2].

Teams who were unsuccessful at implementation in 6 months often needed a longer timeline to overcome barriers.

## Discussion

Although literature on evaluations of implementation and sustainment typically focus on a single evidence-based practice (EBP), learning health systems often rapidly implement and evaluate multiple EBPs simultaneously [34–36]. However, implementing and evaluating multiple EBPs simultaneously is complex, requires significant resources, and is not well understood in the literature to date. More empirical evidence on models of diffusion within learning health systems is needed. Our results help advance the field of implementation science by (1) describing DoE’s “bottom up” approach to engaging employees and providing a process by which to submit their EBPs and involving executive leaders in choosing which EBPs to implement and (2) highlighting methods for comparing implementation and sustainment of diverse EBPs and considering how to strengthen future comparisons of multiple EBPs. The DoE is a unique model that other integrated health systems who desire to become learning organizations can learn from and consider implementing within their own systems.

In the next sections, we describe how our results relate to and extend implementation science literature. First, in our evaluation, we learned that the metrics associated with each GSP varied widely, which made it difficult to identify single measures of implementation and sustainment that could be used to measure outcomes across all GSPs. However, using the CFIR construct ratings and ordinal variables for implementation and sustainment, we were able to align and compare outcomes across 23 diverse practices. When comparing diverse GSPs, implementation support, staffing, and resources were the major factors influencing successful implementation and sustainment. Specifically, DoE’s extensive implementation support resulted in initial implementation success unless the team encountered insurmountable barriers related to obtaining necessary staffing or resources for their GSP. In addition, most teams sustained their practice, unless they were not able to maintain necessary staffing or resources. Two CFIR constructs, *Engaging Key Stakeholders* and *Available Resources*, were key for both initial implementation success and sustainment, which is similar to factors noted in prior literature on programs both within and outside the VHA [37, 38]. Other CFIR constructs did not have a major influence on implementation and sustainment outcomes due to the high level of implementation support provided, which helped teams overcome most barriers.

Second, our results diverge from existing literature on the utility of implementation milestones as predictors of sustainment for diverse EBPs [39–41]. Over half of the teams that did not achieve implementation milestones were able to resolve barriers related to staffing and resources with more time and sustain their practice 1.5 years later. Furthermore, teams that did achieve implementation milestones did not always sustain their GSP; nearly one-third of the teams with successful implementation at 6 months did not sustain their GSP.

Third, our results support a need for more flexible and dynamic assessments of sustainment when comparing diverse EBPs [42, 43]. Although there were differences among teams based on implementation success at 6 months, our anticipated sustainment measure was a more accurate indicator of actual sustainment than implementation milestones. Most teams (81%) that did not achieve 6-month milestones, and slightly more than half of teams (56%) who did achieve 6-month milestones, accurately anticipated prospects for sustainment. It is possible the former group was more accurate in their predictions because they were more aware of barriers. Research on how to simultaneously assess the sustainability of multiple EBPs is needed because it allows organizations to determine if there is a return on investment [34]. However, studying sustainability for multiple EBPs is time intensive, expensive, and requires follow-up beyond the usual scope of funding timelines. Our approach to assessing anticipated sustainment across diverse EBPs may be an appropriate measure that does not require the time or money associated with long-term follow-up. In addition, eliciting feedback prior to the sustainment phase provides an opportunity to intervene and improve the likelihood of success. As our evaluation of DoE evolves, we intend to use a dynamic approach by adding qualitative interviews with implementing teams to improve how we obtain information on anticipated sustainment and sustainment over time.

Fourth, our results prompted us to reconsider the standard “one size fits all” approach to rapid implementation timelines for diverse EBPs; these timelines may be set arbitrarily or are constrained by project funding structures [44]. In our case, the DoE prescribed a 6-month rapid implementation timeline with implementation support. While 6 months was a feasible goal for most teams implementing GSPs, the timeline was unrealistic for teams facing large barriers associated with obtaining staffing and resources. However, it is important to note that teams that did not meet the initial timeline often completed implementation within an additional 1.5 years. Given that the average time to implement EBPs in routine care is 12–17 years, this still represents rapid implementation. Understanding if more flexible or tailored implementation timelines, e.g., based on the readiness of the facility and/or the complexity of implementation, increases implementation and sustainment is an important area for future research.

Fifth, there is a growing need for developing, evaluating, and comparing models for diffusing diverse EBPs across large health systems. Outside of the DoE, there are very few large-scale models of diffusion. Among the few existing models is Kaiser Permanente’s E-SCOPE: Evidence Scanning for Clinical, Operational, and Practice Efficiencies. E-SCOPE has 4 steps: searching the literature quarterly, selecting EBPs to implement, supporting implementation of selected EBPs, and monitoring implementation [6, 7]. In comparison to DoE’s “bottom up” approach to diffusion, E-SCOPE takes a “top down” approach by choosing EBPs from the literature to implement. The extent to which one model of diffusion is more effective than the other (and in which contexts) is a topic for future research. Our results suggest that the aggregate of CFIR construct ratings in combination with ordinal implementation and sustainment outcomes could be used to measure the success of diffusion models, but more research is needed to identify the various facets of measurement, e.g., impact on patients, staff, and facilities, cost-effectiveness.

Sixth, despite the significant changes in DoE’s senior leadership since its creation, it has survived, evolved, and strengthened over time, illustrating the durability of the model. To strengthen the DoE moving forward, we offer the following suggestions: (1) tailor the 6-month timeline, as needed, based on the readiness of the facility and/or the complexity of implementation; (2) enhance Shark Tank bid templates with explicit GSP resource requirements, including resources that must be in place and not simply approved; and (3) elicit anticipated sustainment and offer support to teams expecting significant barriers. Nonetheless, the high level of implementation and sustainment in the DoE suggest that this model is worthy of consideration by other healthcare systems.

### Limitations

This evaluation has several limitations. First, we compared 23 different GSPs that were implemented in 31 unique VHA facilities across the USA, which made this evaluation especially complex. However, we took steps to ensure consistency in the evaluation, including interviewing participants shortly after implementation ended, asking the same interview and survey questions to all teams, using the same interviewers and analysts across cohorts, and analyzing data using well-established CFIR constructs and methods. Though the diversity of GSPs increased complexity of the evaluation, this characteristic also strengthened the evaluation by providing a wider range of innovation characteristics to evaluate across implementation and sustainment outcomes. Second, sustainment survey responses were not as rich as qualitative interviews, leaving some unanswered questions about the reasons why teams did or did not sustain their GSP. However, these limitations are consistent with other literature on how sustainment surveys are time-efficient and cost-effective, but do not provide an in-depth understanding of sustainment [45, 46].

## Conclusions

VHA’s DoE program, which aims to support efforts to implement and sustain diverse evidence-based practices system-wide, can help guide other health systems with the same aim. The high level of implementation and sustainment of GSPs demonstrates that the DoE is a promising large-scale model for other learning health systems seeking to identify and diffuse EBPs, system wide. Initial implementation outcomes did not necessarily guarantee or hinder future sustainment. Results from our evaluation point to novel approaches for assessing and predicting future sustainability.

## Supplementary information

**Additional file 1.** Consolidated Criteria for Reporting Qualitative Studies (COREQ): 32-item checklist.

**Additional file 2.** Interview Guide.

**Additional file 3.** Codebook.

**Additional file 4.** Gold Status Practice Descriptions.

**Additional file 5.** CFIR Construct Ratings.

## Data Availability

The datasets generated and/or analyzed during the current study are not available due to participant privacy but may be available from the corresponding author on reasonable request.

## Abbreviations

CFIR: Consolidated Framework for Implementation Research
DoE: Diffusion of Excellence
GSP: Gold Status Practice
EBP: Evidence-based practice
VHA: Veterans Health Administration
