# Supplementary material for: Implementation and sustainment of diverse practices in a large integrated health system: a mixed methods study
Source: Implement Sci Commun. 2020 Jul 3;1:61. doi: 10.1186/s43058-020-00053-1 (PMC7427879; doi:10.1186/s43058-020-00053-1)
Supplement: Supplementary file 2 — Additional file 2. Interview Guide. [file 43058_2020_53_MOESM2_ESM.docx]

**Additional file 2. Interview Guide**

| **CFIR Construct** | **Question** | **Probe(s)** |
| --- | --- | --- |
| Role in Facility | - What is your role at your site outside of implementation? - What is your role with implementation? | - Length of VA career |
| Innovation Characteristics | - How would you describe **[practice]**? | - What is the ultimate aim of the practice? |
| Multiple  Tension for Change (TfC) | - Do you know why your facility/VISN director chose to bid on this practice? | - What did you/others think about this decision? - What level of need did you/others see for the practice? |
| Engaging (ENG FIL) | - How did you learn that your facility/VISN director (Shark) had bid on the practice? - How did you become involved with implementation? | - Were you invited to help choose which practices to bid on and/or to participate in the bidding?   - Note: The Shark was supposed to schedule a conversation with them directly to discuss:     - Their role as the Implementing Fellows     - The resources bid during Shark Tank that they will utilize for implementation - Were you the right person to lead implementation?   - Experience?   - Skills?   - Time? Collateral or dedicated?   - Authority? |
| Engaging: Key Stakeholders (ENG KS)  External Change Agent (ECA) | - Who else was involved with implementation? - What was the role of ____________? - What was most helpful about support from the DEI Team Leads (Atlas)? - Was anything unhelpful about their support? - What was most/least helpful about support from the GSFF? - Was anything unhelpful about their support? | - Onsite   - Colleagues   - Innovation Specialist (Innovator’s Network Role)   - Leadership     - Did the facility/VISN director (Shark) provide the bid resources/other support during implementation?     - Did the bid resources help? - Offsite   - DEI Team Lead   - Gold Status Facility Fellow   - Other Implementing Facility Fellow |
| External Change Agent (ECA) | - What might have happened if you hadn’t had support from the action team? | - What happened/will happen after you lost support from the action team at ~6 months? |
| Multiple | - Can you tell me about your experience generally with implementing **[practice]**? |  |
| Multiple | - What hindered implementation at your site? - What strategies did you use to overcome barriers? - What helped implementation at your site? | - Are there any barriers that you could not overcome? |
| Multiple  Planning (PLAN) | - What was your experience like at the DEI Summit? | - Were you able to complete the pre-summit homework assignment? - How was the Action Planning process? - How did you think your practice compared to others at the summit? |
| Compatibility (COMP) | - How well did the practice fit with existing work processes at your site? - Did you have all the necessary infrastructure (e.g., staff, processes, policies) in place to implement this practice? | - Did you have anything similar to the practice already in place? - Were there any advantages/disadvantages to the practice compared to the old process? |
| Relative Priority (RP) | - How did the priority of implementing the practice compare to other priorities in your organization? | - For staff? - For leadership? |
| Key Stakeholders (ENG KS) | - Tell me how you engaged VA staff. | - Communication? - Materials? |
| Key Stakeholders (ENG KS) | - Were you able to bring everyone you needed on board?? | - Barriers to engaging VA staff? - Facilitators to engaging VA staff? |
| Innovation Participants (ENG IS) | - Describe how you engaged Veterans - Did you receive any feedback from Veterans? | - How well is the practice meeting the needs of Veterans? |
| Adaptations (ADPT) | - What adaptations did you make to the practice? | - Why did you make these adaptions? - What did the Gold Status Fellow think about these adaptations? |
| Reflecting and Evaluating (RE) | - What kind of information did you collect during implementation about the status and progress of implementation (not the practice)? - How was this information used? | - Any information that you did not collect, but would have been useful? |
| Implementation Success | - Overall, from a scale of 1 to 10, where 1 is unsuccessful, and 10 is successful, how would you rate your site in implementing this practice? Why? | - What would be needed to increase that number? |
| Sustainability | - How likely is it that the practice will continue to be used at your site? Why? |  |
| Multiple | - What advice would you give to other sites that are implementing this practice? - What suggestions do you have for future Shark Tanks? - Is there anything else you feel is relevant for us to know about? |  |
