# Supplementary material for: Implementation and sustainment of diverse practices in a large integrated health system: a mixed methods study
Source: Implement Sci Commun. 2020 Jul 3;1:61. doi: 10.1186/s43058-020-00053-1 (PMC7427879; doi:10.1186/s43058-020-00053-1)
Supplement: Supplementary file 3 — Additional file 3. Codebook. [file 43058_2020_53_MOESM3_ESM.docx]

**Addititional file 3. Codebook**

**Diffusion of Excellence Cosolidated Framework for Implementation Research Codebook**

**Definitions**

| 1. **Innovation Characteristics** |  |
| --- | --- |
| 1. Innovation Description (DESCRIPT) | Definition: Description of the Gold Status Practice (GSP) and GSP components, as well as the history of the development of the GSP and ultimate aim of the GSP.  Inclusion Criteria: Include statements re: differences in how Implementing Facilities and Gold Status Facilities conceptualized a GSP.  Include statements re: what resources are needed to implement the GSP, e.g., a resource neutral GSP, a GSP that requires X resource.  Double code statements re: the GSP being adaptable by nature, i.e., a process improvement intervention that was designed to be used in any service, to DESCRIPT and ADPTY.  Exclusion Criteria: This code is typically mutually exclusive from CFIR codes; the only time you would also code CFIR is if the Implementing Facility is specifically referenced when asking about the GSP. |
| 1. Innovation Source (IS) | Definition: Perception of key stakeholders about whether the GSP is externally or internally developed.  Inclusion Criteria: Include statements re: the source of the GSP and the extent to which interviewees view the change as internal to the organization, e.g., an internally developed program, or external to the organization, e.g., a program coming from the outside. Note: May code and rate as "I" for internal or "E" for external.  Note: It is likely all participant will view the GSP as external, because it is being implemented through the DoE Shark Tank process.  Exclusion Criteria: Exclude or double code statements re: who participated in the decision process to implement the GSP to Engaging, as an indication of early (or late) engagement and Leadership Engagement: Bidding. Participation in decision-making is an effective engagement strategy to help people feel ownership of the innovation. |
| 1. Evidence Strength & Quality (ESQ) | Definition: Stakeholders’ perceptions of the quality and validity of evidence supporting the belief that the GSP will have desired outcomes.  Inclusion Criteria: Include statements re: awareness of evidence and the strength and quality of evidence, as well as the absence of evidence or a desire for different types of evidence, such as pilot results instead of evidence from the literature.  Exclusion Criteria: Exclude statements re: the receipt of evidence as an engagement strategy to Engaging: Key Stakeholders. |
| 1. Relative Advantage (RA) | Definition: Stakeholders’ perception of the advantage of implementing the GSP versus an alternative solution.  Inclusion Criteria: Include statements re: the GSP being better (or worse) than existing programs or the status quo.  Note: TFC and RA can co-occur if one is present and one is absent, i.e., they see the RA of something but not the TFC.  Include statements re: the financial or resource benefit to the facility from the GSP, i.e., the Cost-Effectiveness of a GSP or a GSP that provides high value care (using resources properly to create a good product and/or have good outcomes).  Exclusion Criteria: Exclude statements re: a strong need for the GSP and/or that the current situation is untenable and code to Tension for Change. |
| 1. Adaptability (ADPTY) | Definition: The degree to which an GSP can be adapted, tailored, refined, or reinvented to meet local needs.  Inclusion Criteria: Include statements re: the (in)ability to adapt the GSP to their context, e.g., complaints about the rigidity of the protocol.  Double code statements re: the GSP being adaptable by nature, i.e., a process improvement intervention that was designed to be used in any service, to DESCRIPT and ADPTY.  Exclusion Criteria: Exclude statements re: adaptations to the GSP and code to ADPTS. |
| 1. Trialability (TRIAL) | Definition: The ability to test the GSP on a small scale in the organization, and to be able to reverse course (undo implementation) if warranted.  Inclusion Criteria: Include statements re: whether the facility piloted the GSP in the past or has plans to in the future, and comments about whether they believe it is (im)possible to conduct a pilot.  Exclusion Criteria: Exclude descriptions of use of results from local or regional pilots to Evidence Strength & Quality. |
| 1. Complexity (CMPX) | Definition: Perceived difficulty of the innovation, reflected by duration, scope, radicalness, disruptiveness, centrality, and intricacy and number of steps required to implement.  Inclusion Criteria: Code statements re: the complexity of the GSP itself (which persists after implementation), the decision-making options and/or number of decision steps.  Exclusion Criteria: Exclude statements re: the complexity of implementation and code to the appropriate CFIR code, e.g., difficulties re: space are coded to Available Resources and difficulties re: engaging participants in a new program are coded to Engaging: Innovation Participants. |
| 1. Design Quality & Packaging (DQP) | Definition: Perceived excellence in how the GSP is bundled, presented, and assembled.  Inclusion Criteria: Include statements re: the quality of the materials and packaging used for the innovation. Include online or computer-based materials, systems, and user interfaces.  Include statements re: Shark Tank Pitch videos and PPTs as well as materials shared from the GSF.  Exclusion Criteria: Exclude statements re: the presence or absence of materials and code to Available Resources.  Exclude statements re: the receipt of materials as an engagement strategy and code to Engaging. |
| 1. Cost (COST) | Definition: Costs of the GSP and costs associated with implementing the GSP including investment, supply, and opportunity costs.  Inclusion Criteria: Include statements re: the cost of the GSP and its implementation.  Exclusion Criteria: Exclude statements re: physical space and time, and code to Available Resources. |
| 1. **Outer Setting** | Definition: The setting outside of the Implementing Facility or Implementing VISN. |
| 1. Patients Needs and Resources (PNR) | Definition: The extent to which patient (customer) needs (and desires/preferences) drive adoption, implementation, and/or adaptations to the targeted innovation.  Inclusion Criteria: Include statements re: a belief (or awareness) that patients will like/be satisfied by the GSP.  Note: Relationship code PNR > TFC when applicable.  Exclusion Criteria: Exclude statements re: participant feedback to staff on the innovation, i.e., satisfaction and success in a program, i.e., "They love coming and they always come," is ENG: IP VETS. |
| 1. Cosmopolitanism (COSMO) | Definition: The degree to which an organization is networked with other external organizations.  Inclusion Criteria:  Include statements re: information sharing and co-learning between 1. The GSF and IF(s); 2. The IFs (when there is more than one that wins the bid); and 3. The IF(s) and facilities implementing the GSP outside the DoE, i.e., quality collaboratives.  Include statements re: adaptations made at the IF circling back to the GSF.  Exclusion Criteria: Exclude statements re: general networking, communication, and relationships within the organization such as; descriptions of meetings, email groups, or other methods of keeping people connected and informed, and statements re: team formation, quality, and functioning, and code to Networks & Communications. |
| 1. Peer Pressure (PP) | Definition: Mimetic or competitive pressure to implement an innovation, typically because most or other key peer or competing organizations (within or outside of the local geographic area) have already implemented or are in a bid for a competitive edge.  Inclusion Criteria: Include statements re: acting to “keep up with the Joneses” down the street (literally or figuratively) and close the gap (whether gap is actual or perceived). |
| 1. External Policy & Incentives (EPI) | Definition: A broad construct that includes external strategies to spread GSPs, including policy and regulations (governmental or other central entity), external mandates, recommendations and guidelines, pay-for-performance, collaboratives, and public or benchmark reporting.  Inclusion Criteria: Include statements re: national or VISN-level (external) performance measures (PMs) leading to the GSP being selected for implementation in subcode: EPI: PM.  Note: Relationship code EPI: PM > G&F and EPI: PM > TFC when applicable.  Include statements re: the local or national unions in subcode: EPI: UNION.  Include statements re: centralized decision making to EPI: CDD.  Note: Relationship code EPI: CMD \| ENG KS when it delays/stops hiring.  Exclusion Criteria: Service- or section-level policies that are not external to local setting. |
| 1. Leadership Engagement (from the Outer Setting)   (EXT LE) | Definition: The involvement and support from national or VISN level leadership, e.g., leadership in the Outer Setting.  Inclusion Criteria: Include statements re: EXT LE attending calls and helping overcome barriers or partnering as a national office. |
| 1. DOE | Definition: A broad construct that includes any information re: DoE as an external innovation.  Inclusion Criteria: Include statements re: what’s working and what’s not working as well as recommendations re: DoE processes and activities.  Include statements re: how DoE influenced implementation, i.e., national attention from DoE increased the priority of implementation.  Include statements re: DoE Leadership intervention, i.e., when DoE leadership stepped in to problem-solve.  Exclusion Criteria: Exclude statements re: bidding and code to LE: BID and relevant ENG KS.  Exclude statements re: Summit/Base Camp unless they have feedback about the Summit/Base Camp (i.e., not attendance only).  Exclude statements re: homework unless they have feedback on the homework (i.e., not completion only).  Exclude statements re: weekly meetings unless they have feedback about the meetings (i.e., not attendance only).  Exclude statements that are coded to ECA: ESP. |
| 1. **Inner Setting** | Definition: The setting within the Implementing Facility or Implementing VISN.  A-D exist outside of the implementation; E/F are specific to the implementation. |
| 1. Staff Needs & Resources (SNR) | Definition: The extent to which staff (key stakeholders) needs (and desires/preferences) drive adoption, implementation, and/or adaptations to the targeted innovation.  Note: This code is the equivalent to the PNR code in the Outer Setting, except it’s for staff in the Inner Setting.  Inclusion Criteria: Include statements re: a belief (or awareness) that staff will like/be satisfied by the GSP.  Include statements re: all levels and types of staff, e.g., clinical or administrative.  Note: Relationship code SNR > TFC when applicable.  Exclusion Criteria: Exclude statements re: participant feedback, i.e., satisfaction and success in a program, i.e., "Staff enjoy the new process,” to the relevant ENG code: INT KS or IP STAFF. |
| 1. Structural Characteristics (SC) | Definition: The social architecture, age, maturity, and size of an organization.  Inclusion Criteria: Include statements re: how geographical location influences context and implementation, i.e., rural or urban.  Include statements re: turnover and attrition. |
| 1. Networks & Communications (NC) | Definition: The nature and quality of webs of social networks, and the nature and quality of formal and informal communications within an organization.  Inclusion Criteria: Include statements re: general networking, communication, and relationships in the organization, such as; descriptions of meetings, email groups, or other methods of keeping people connected and informed, and statements re: team formation, quality, and functioning.  Exclusion Criteria: Exclude statements re: implementation leaders' and users' access to knowledge and information re: using the program, i.e., training on the mechanics of the program and code to Access to Knowledge & Information.  Exclude statements re: engagement strategies and outcomes, e.g., how key stakeholders became engaged with the GSP and what their role is in implementation, and code to ENG KS.  Exclude descriptions of outside group memberships and networking done outside the organization and code to Cosmopolitanism. |
| 1. Culture (CULT) | Definition: Norms, values, and basic assumptions of a given organization.  Inclusion Criteria: Inclusion criteria, and potential sub-codes, will depend on the framework or definition used for “culture.”  Include statements re: having a “culture of innovation” or a culture “resistant to change.” |
| 1. Implementation Climate (IC REC) | Definition: The absorptive capacity for change, shared receptivity of involved individuals to an innovation, and the extent to which use of that GSP will be rewarded, supported, and expected within their organization.  Exclusion Criteria: Exclude statements re: the general level of receptivity that are captured in the sub-codes.  Exclude statements re: the general level of receptivity that are specific to a key role in implementation, i.e., leadership or any role under ENG. |
| 1. Tension for Change (TFC) | Definition: The degree to which stakeholders perceive the current situation as intolerable or needing change.  Inclusion Criteria: Include statements re: the level of need for the GSP and/or the current situation being untenable, e.g., statements re: GSP being necessary or the GSP being redundant with other programs. Note: If a participant states that the GSP is redundant with a preferred existing program, (double) code lack of Relative Advantage, see exclusion criteria below.  Note: TFC and RA can co-occur if one is present and one is absent, i.e., they see the RA of something but not the TFC.  Exclusion Criteria: Exclude statements re: the aim of the GSP and code to DESCRIPT when it’s re: the general aim of the GSP, rather than the need in the facility. |
| 1. Compatibility (COMP VAL; COMP WP) | Definition: The degree of tangible fit between meaning and values attached to the GSP by involved individuals, how those align with individuals’ own norms, values, and perceived risks and needs, and how the GSP fits with existing workflows and systems.  Inclusion Criteria:  Include statements re: compatibility with values to subcode COMP: VAL.  Include statement re: compatibility with work processes to subcode COMP: WP.  Include statements re: how existing or lacking processes and/or infrastructure influence implementation via COMP WP; this sometimes manifests as “scope creep” for implementation, because necessary work processes were not existing prior to implementation; double code to ADPTs when the participant perceives these changes as part of the GSP. This may also sometimes capture issues re: a different “conceptualization.” |
| 1. Relative Priority (RP) | Definition: Individuals’ shared perception of the importance of the implementation within the organization.  Inclusion Criteria: Include statements re: the relative priority of implementation, e.g., statements re: change fatigue in the organization due to implementation of many other programs.  Note: This is ORGANIZATIONAL relative priority; high RP for the FIL/IFF is not necessarily a reflection of organizational RP. |
| 1. Organizational Incentives & Rewards (OIR) | Definition: Extrinsic incentives such as goal-sharing, awards, performance reviews, promotions, and raises in salary, and less tangible incentives such as increased stature or respect.  Inclusion Criteria: Include statements re: whether organizational incentive systems are in place to foster (or hinder) implementation, e.g., rewards or disincentives for staff engaging in the innovation.  Exclusion Criteria: General incentives and rewards not specifically re: practices linked to implementation (e.g., tuition reimbursement). |
| 1. Goals & Feedback (GF) | Definition: The degree to which goals are clearly communicated, acted upon, and fed back to staff, and alignment of that feedback with goals.  Inclusion Criteria:  This construct seeks to understand 1) if the facility is/is not data driven (this is a characteristic of the organization, not an implementation process, i.e., it's true of the organization regardless of any implementation efforts) and 2) if the targeted GSP is/is not in line with organizational goals. In other words, GF is a characteristic of the organization, but it's also proximal to the topic addressed by the targeted GSP (vs. being broader, which would speak to Culture or Structural Characteristics).  Exclusion Criteria: Exclude statements re: setting goals specific to implementation and code to PLAN (as an implementation process).  Exclude statements re: data collection and monitoring progress towards implementation goals to RE (as an implementation process).  Exclude statements re: performance measures that come down from above to EPI: PM, unless the PMs have been owned/internalized by the facility (and are in line with the innovation). |
| 1. Learning Climate (LC) | Definition: A climate in which: 1. Leaders express their own fallibility and need for team members’ assistance and input; 2. Team members feel that they are essential, valued, and knowledgeable partners in the change process; 3. Individuals feel psychologically safe to try new methods; and 4. There is sufficient time and space for reflective thinking and evaluation.  Inclusion Criteria: Include statements re: the degree to which key components of an organization exhibit a “learning climate.” |
| 1. Readiness for Implementation | Definition: Tangible and immediate indicators of organizational commitment to its decision to implement an innovation.  Inclusion Criteria: Include statements re: the general level of readiness for implementation.  Exclusion Criteria: Exclude statements re: the general level of readiness for implementation that are captured in the sub-codes. |
| 1. Leadership Engagement (from the Inner Setting) (LE BID; LE IMP) | Definition: Commitment, involvement, and accountability of leaders and managers with the implementation of the innovation.  Inclusion Criteria: Include statements re: the director bidding on the GSP to LE BID, i.e., did they follow the guidelines from DOE.  Include statements re: the director’s level of involvement during implementation to LE IMP.  Include statements re: mid-level leadership’s level of involvement during implementation to ML LE.  Note: When the unit of analysis is the VISN: The VISN director is the "high level" of leadership and the Facility Directors (and Service Chiefs) are the ML LE. When the unit of analysis is the facility: The Facility director is the "high level" of leadership and the Service Chiefs are the ML LE. If the VISN director was referenced here; s/he would be EXT LE. |
| 1. Available Resources (AR) | Definition: The level of organizational resources dedicated for implementation and on-going operations including physical space and time (protected FTE/time).  Inclusion Criteria: Include statements re: the presence or absence of resources specific to the GSP that is being implemented.  Note: AR is about presence/absence of materials (both re: the targeted GSP (e.g., program manual) and the environment (e.g., time, space, IT). DQP is about quality of materials that are specific to the targeted innovation, whereas quality of environmental resources (e.g., a bad classroom) is still coded to AR.  Exclusion Criteria: Exclude statements re: training and education and code to AKI.  Exclude statements re: the quality of materials and code to DQP. |
| 1. Access to Knowledge & Information (AKI) | Definition: Ease of access to digestible information and knowledge about the GSP and how to incorporate it into work tasks.  Inclusion Criteria: Include statements re: implementation leaders' and users' access to knowledge and information re: use of the program, i.e., training on the mechanics of the program.  Note: AKI is typically related to the IFF’s AKI from the GSF or staff AKI from the IFF.  Exclusion Criteria: Exclude statement re: lack of information about DEE generally.  Exclude statements re: general networking, communication, and relationships in the organization, such as descriptions of meetings, email groups, or other methods of keeping people connected and informed, and statements re: team formation, quality, and functioning, and code to Networks & Communications. |
| 1. **Characteristics of Individuals** | Code information about individuals to their role code under ENG. |
| 1. **Process** | The intent of this domain is to assess the presence and quality of these constructs, not to be prescriptive. In other words, these constructs are designed to be in alignment with the best frameworks. |
| 1. Planning (PLAN) | Definition: The degree to which a scheme or method of behavior and tasks for implementing an GSP are developed in advance, and the quality of those schemes or methods.  Inclusion Criteria: Include statements re: creating an Action Plan at Summit/Basecamp or during weekly calls.  Include statements re: setting goals for implementation  Include evidence of pre-implementation diagnostic assessments and planning, as well as refinements to the plan. Activities that are codable under Planning include: Context/needs assessment, creating a plan, planning/developing PDSA cycles, setting implementation goals (see OCM), planning pilots/incremental approaches, etc.  Exclusion Criteria: Exclude statements re: collecting data for the purpose of RE (even if they haven’t completed the process) and code to RE. |
| 1. Implementation Strategies (IMP STRAT) | Definition: The different strategies used by ECA and/or the FIL/IFF to implement the GSP at the IF, **outside of what is captured in CFIR: Planning, Engaging, and Reflecting and Evaluating.** Note: May later be mapped to ERIC.  Inclusion Criteria: Include statements re: phased implementation efforts. |
| 1. Adaptations (ADPT) | Definition: Adaptations made to the GSP. Note: May later be mapped to the Wiltsey-Stirman Adaptations Framework.  Inclusion Criteria: Suggestions for improvement or adaptation can be captured in this code.  Include statements re: perceived adaptations re: “scope creep” of the GSP; see COMP WP. This may also sometimes capture issues re: a different “conceptualization.”  Exclusion Criteria:  Exclude statements re: the adaptability of the GSP and code to ADPTY. |
| 1. Engaging | Definition: Attracting and involving appropriate individuals in the implementation and use of the GSP through a combined strategy of social marketing, education, role modeling, training, and other similar activities.  Inclusion Criteria: Include statements re: engagement strategies and outcomes, i.e., if and how staff and participants became engaged with the GSP and what their role is in implementation.  Include statements re: the "quality" of staff - their capabilities, motivation, and skills, i.e., how good they are at their job.    Include statements re: the inability to ENG staff (FIL, KS, etc.) due to the inability to hire in a timely matter.  Note: Relationship code EPI: CMD \| ENG KS when it delays/stops hiring.  Exclusion Criteria: Exclude or double code statements re: who participated in the decision process to implement the GSP to Innovation Source, as an indicator of internal or external GSP source. |
| 1. Opinion Leaders (OL) | Definition: Individuals in an organization that have formal or informal influence on the attitudes and beliefs of their colleagues with respect to implementing the innovation. |
| 1. Formally Appointed Internal Implementation Leaders (FIL) (IFF) | Definition: Individuals from within the organization who have been formally appointed with responsibility for implementing an GSP as coordinator, project manager, team leader, or other similar role.  Inclusion Criteria: Include statements re: the FILs role in the facility and if they were right for the job of implementation. Also include the FILs receptivity to implementation.  Double code statements re: their time available for implementation, e.g., it was a collateral duty, or they were given protected time, to Available Resources. |
| 1. Champions (CHAMP) | Definition: “Individuals who dedicate themselves to supporting, marketing, and ‘driving through’ an [implementation]”, overcoming indifference or resistance that the GSP may provoke in an organization. |
| 1. External Change Agents (ECA: ESP; ECA GSFF) | Definition: Individuals who are affiliated with an outside entity who formally influence or facilitate GSP decisions in a desirable direction.  Inclusion Criteria: Include statements re: the External Support Providers in subcode ECA: ESP.  Include statements re: the Gold Status Facility Fellow to in subcode ECA: GSFF. |
| 1. Key Stakeholders (ENG KS IMP; ENG KS INT) | Definition: Individuals from within the organization that are directly impacted by the innovation.    Inclusion Criteria: Include statements re: the KS that are responsible for implementation in subcode ENG KS IMP (i.e., their role may end when implementation is complete)  Include statement re: the KS that are responsible for administering and/or sustaining the GSP in subcode ENG KS INT (i.e., their role will start or continue after implementation is complete).  Note: Some ENG KS IMP may also be ENG KS INT, i.e., they are responsible for implementation and later administration/sustainment of the GSP. |
| 1. Innovation Participants (ENG IP VET; ENG IP STAFF) | Definition: Individuals served by the GSP, e.g., patients in a prevention program in a hospital, staff in a training program.  Inclusion Criteria: Include statements re: engaging Veterans in the GSP (a clinical intervention) in subcode ENG IP VET.  Include statements re: engaging staff in the GSP (a staff intervention) in subcode ENG IP STAFF. Note: This code is only relevant with staff when the GSP is some type of program/event/training designed for staff. |
| 1. Executing (EXC) | Definition: Carrying out or accomplishing the implementation according to plan.  Inclusion Criteria: Include statements re: how implementation occurred with respect to the implementation plan. Note: Executing is coded very infrequently due to a lack of planning. However, some studies have used fidelity measures to assess executing, as an indication of the degree to which implementation was accomplished according to plan. |
| 1. Reflecting & Evaluating (RE IMP; RE INT) | Definition: Quantitative and qualitative feedback about the progress and quality of implementation accompanied with regular personal and team debriefing about progress and experience.  Inclusion Criteria: Include statements re: reflecting and evaluating on the implementation process, i.e., what’s working/what’s not working and tracking progress of implementation in subcode RE IMP.  Include statements re: reflecting and evaluating on the effectiveness of the intervention, i.e., is the GSP working or not in subcode RE INT.  Exclusion Criteria: Exclude statements re: the (lack of) alignment of implementation and GSP goals with larger organizational goals, as well as feedback to staff re: those goals, e.g., regular audit and feedback showing any gaps between the current organizational status and the goal, and code to Goals & Feedback.  Exclude statements re: reflecting and evaluating that participants may do during the interview, for example, re: the success of the implementation or the GSP and code to Outcomes. |
| 1. **Outcomes** |  |
| 1. Implementation Success (IMP SUC) | Definition: Perception of the success of GSP implementation at the IF.  Inclusion Criteria: Include statements re: the timeliness of implementation (e.g., delays) and the level of completion of implementation. |
| 1. Intervention Success (INT SUC) | Definition: Perception of the success or effectiveness of the GSP at the IF. |
| 1. Sustainability (SUSTAIN) | Definition: Perception of if the GSP will be sustained at the IF. |
| 1. Diffusion (DIFFUSE) | Definition: Perception of if the GSP will be diffused nationally.  Inclusion Criteria: Include statements re: why the GSP was chosen for national roll-out, and criteria for national diffusion, e.g., GSP maturity, program office uptake and ownership, system wide need. |
