# Supplementary material for: Implementation and sustainment of diverse practices in a large integrated health system: a mixed methods study
Source: Implement Sci Commun. 2020 Jul 3;1:61. doi: 10.1186/s43058-020-00053-1 (PMC7427879; doi:10.1186/s43058-020-00053-1)
Supplement: Supplementary file 4 — Additional file 4. Gold Status Practice Descriptions. [file 43058_2020_53_MOESM4_ESM.docx]

| **Additional file 4. Gold Status Practice Descriptions** | | |
| --- | --- | --- |
| **Cohort 2 Gold Status Practice Descriptions** | | |
| **Category** | **Name** | **Short Description** |
| Access | Home-Based Mental Health Evaluation (HOME) Program | This practice provides clinical services during the transition from inpatient to outpatient mental health care. |
| Access | National TeleWound Care Practice | This practice provides expedited remote diagnosis and treatment of various types of wounds via clinical video telehealth, e-Consults, and Store-and-Forward telehealth. |
| Access | Improving Access through Consult Triage | This practice requires physicians to review consults at the time of the order request and triage patients appropriately. |
| Care Coordination | VHA Rapid Naloxone | This practice expands the availability of Narcan Nasal Spray by distributing it to VA Police and Veterans, and putting it within some Automated External Defibrillator (AED) cabinets. |
| Care Coordination | Women's Health Mini-Residency | This practice uses a pre-set curriculum from the Office of Women's Health Education to increase the volume of clinicians competent to care for female Veterans. |
| Employee Engagement | Competency Tracker Program | This practice allows users to track employee performance, quickly identify competent coverage for call-ins/no-shows and employees on leave, and assess new areas to focus training using a custom board. |
| Employee Engagement | Jump Start Program for New Employees | This practice establishes a dedicated new hire welcome website and the expansion of the facility-coaching program to include matching new hires with experienced coaches. |
| Employee Engagement | Veterans Engagement Day | This practice offers staff, who do not normally interact with Veterans, the opportunity to spend a day meeting Veterans and hearing their stories. |
| Quality and Safety | Novel Screening and Transfer Process for Patients Seeking Substance Detoxification | This practice uses an enhanced screening and transfer algorithm (based on available literature and multidisciplinary consensus) that is embedded in a novel E-consultation order set for providers to use with patients seeking substance detoxification. |
| Quality and Safety | Project HAPPEN: Hospital Acquired Pneumonia Prevention by Engaging Nurses | This practice engages nursing staff to provide oral care to Veterans. |
| **Cohort 2 Gold Status Practice Descriptions Continued** | | |
| **Category** | **Name** | **Short Description** |
| Veterans Experience | Chaplain Groups for Veterans with Moral Injury | This practice is a group visit for Veterans diagnosed with Posttraumatic Stress Disorder (PTSD) and struggling with service-connected moral injury. |
| Veterans Experience | My Life, My Story | This practice involves interviewing Veterans to capture their life story and adding it to their VA Electronic Health Record for VA providers to read. |
| Veterans Experience | Pain University | This practice is a comprehensive university-like program that offers ~30 classes and treatment labs to Veterans related to pain. |
| **Cohort 3 Gold Status Practice Descriptions** | | |
| **Category** | **Name** | **Short Description** |
| Access | A Best Practice: The Collaborative Consult Scheduling Model | This practice initiates a partnership between services to create a dedicated team of Advanced Medical Support Assistants (AMSAs) to focus on consult scheduling; the team can make one call and address several consults for a single Veteran. |
| Access | VistA Automation for Prosthetics | This practice automates the home oxygen billing process by integrating Microsoft Excel and Veterans Information Systems and Technology Architecture (VistA); staff no longer need to print the bill and complete lengthy reviews, allowing for employees to improve access elsewhere in Prosthetics. |
| Care Coordination | HBPC Interdisciplinary Project to Reduce Hospitalization of Veterans with COPD, CHF, and Pneumonia | This practice uses a multidisciplinary team to decrease hospitalizations by educating caregivers and patients on disease management using Zone Charts and coordinating care with community providers. |
| Care Coordination | VIONE | This practice is a model for clinical pharmacists to review each patient's medication profile to identify appropriate medications to de-prescribe. |
| Quality and Safety | SharePoint Construction Safety Tool for All VHA Facilities | This practice provides an electronic means to document construction safety inspections using SharePoint; the tool provides a mechanism to track deficiencies from identification to corrective action, ensuring compliance with federal regulations. |

| **Cohort 3 Gold Status Practice Descriptions Continued** | | |
| --- | --- | --- |
| **Category** | **Name** | **Short Description** |
| Quality and Safety | Substance Use and Suicide Prevention Group Therapy Module | This practice is a one-hour psychoeducational group therapy session where a SUD treatment specialist and suicide prevention coordinator cover the prevalence of suicide in SUD treatment populations. |
| Veterans Experience | Community Housing Fair | This practice is a “one-stop shop” event to help homeless Veterans achieve permanent housing status in less than eight hours. |
| Veterans Experience | FLOW3 | This practice is a workflow management system that incorporates three custom-designed features to address issues with the process for authorization of artificial limbs: an artificial limb consult template, a consult comment tool, and a web-based dashboard with custom screens for staff member workflow management. |
| Veterans Experience | Partnering with Veteran Service Agencies and Faith-Based Organizations to Prevent Veteran and Service Member Suicides | This practice trains clergy and their congregations to identify and refer at-risk Veterans and Service Members to VA care. |
| Veterans Experience | VeTRANS - Veteran Transportation Project | This practice is a collaborative solution between Voluntary Service, Social Work Service, and Health Administration Service that provides transportation to Veterans that are discharged from acute care or the emergency room and do not qualify for Beneficiary Travel or have a viable form of transportation. |
