# Supplementary material for: Implementation and sustainment of diverse practices in a large integrated health system: a mixed methods study
Source: Implement Sci Commun. 2020 Jul 3;1:61. doi: 10.1186/s43058-020-00053-1 (PMC7427879; doi:10.1186/s43058-020-00053-1)
Supplement: Supplementary file 5 — Additional file 5. CFIR Construct Ratings. [file 43058_2020_53_MOESM5_ESM.docx]

## ** Additional file 5: Consolidated Framework for Implementation Research (CFIR) Construct Ratings**
